# Loss of miR-936 leads to acquisition of androgen-independent metastatic phenotype in prostate cancer

**DOI:** 10.1038/s41598-022-20777-5

**Published:** 2022-10-12

**Authors:** Sarathkumar Edachery, Prakash Patil, Rajashekar Mohan, Bhuvanesh Aradhya, Jayaprakash Shetty, Shama Prasada Kabekkodu, Manas Kumar Santra, Sathisha Jayanna Gonchigar, Praveenkumar Shetty

**Affiliations:** 1 Department of Biochemistry, Kuvempu University, Shankaraghatta, Karnataka 577451 India; 2 Division of Proteomics and Cancer Biology, Nitte University Center for Science Education and Research, Nitte (Deemed to be University), Mangaluru, Karnataka 575018 India; 3 Central Research Laboratory, K S Hegde Medical Academy, Nitte (Deemed to be University), Deralakatte, Mangaluru 575018 India; 4 Department of Surgery, All India Institute of Medical Sciences, Mangalagiri, Andhra Pradesh 522503 India; 5 HCG-Suchirayu Hospital, Gokul Road, Hubli, 580030 India; 6 Department of Pathology, K S Hegde Medical Academy, Nitte (Deemed to be University), Deralakatte, Mangaluru 575018 India; 7 School of Life Sciences, Life Science Center, Manipal University, Manipal, 576104 India; 8 National Center for Cell Sciences, Ganesh Kind, Pune, 411007 India; 9 Department of Biochemistry, K S Hegde Medical Academy, Nitte (Deemed to be University), Deralakatte, Mangaluru 575018 India

**Keywords:** Cancer, Molecular biology, Oncology

## Abstract

Prostate cancer (PCa) progresses from a hormone-sensitive, androgen-dependent to a hormone-refractory, androgen-independent metastatic phenotype. Among the many genes implicated, ANXA2, a calcium-dependent phospholipid binding protein, has been found to have a critical role in the progression of PCa into more invasive metastatic phenotype. However, the molecular mechanisms underlying the absence of ANXA2 in early PCa and its recurrence in advanced stage are yet unknown. Moreover, recent studies have observed the deregulation of microRNAs (miRNAs) are involved in the development and progression of PCa. In this study, we found the down-regulation of miR-936 in metastatic PCa wherein its target ANXA2 was overexpressed. Subsequently, it has been shown that the downregulation of miRNA biogenesis by siRNA treatment in ANXA2-null LNCaP cells could induce the expression of ANXA2, indicating the miRNA mediated regulation of ANXA2 expression. Additionally, we demonstrate that miR-936 regulates ANXA2 expression by direct interaction at coding as well as 3′UTR region of ANXA2 mRNA by luciferase reporter assay. Furthermore, the overexpression of miR-936 suppresses the cell proliferation, cell cycle progression, cell migration, and invasion abilities of metastatic PCa PC-3 cells in vitro and tumor forming ability in vivo*.* These results indicate that miR-936 have tumor suppressor properties by regulating the over expression of ANXA2 in hormone-independent metastatic PCa. Moreover, our results suggest that this tumor suppressor miR-936 could be developed as a targeted therapeutic molecule for metastatic PCa control and to improve the prognosis in PCa patients.

## Introduction

Prostate cancer (PCa) is the second most leading cancer in males with an incidence and mortality rates of 30.7 and 7.7 cases per lakh age-standardized individuals, respectively (GLOBOCON 2020). Looking at the current trend of life expectancy rates and the existing age-specific incidence, morbidity, and fatality rates, PCa will become a far greater public health burden in the future. PCa develops in stages, including prostatic intraepithelial neoplasia (PIN), prostate cancer in situ, and hormone-dependent and -independent metastatic disease. The transition of PCa from hormone-dependent to -independent may be due to the loss of EGFR regulation and its altered signaling^1–3^. The increased EGFR expression in combination with functional loss of PTEN (haploinsufficiency, mutation, and deletion) leads to activation of EGFR downstream signalling, particularly the PI3K-AKT pathway, in hormone-independent metastatic PCa^4,5^. This signaling has been linked to the PCa progression to invasion and metastasis. In addition to altered expression of EGFR and PTEN, over expression of calcium-dependent phospholipid-binding protein Annexin A2 (ANXA2) is the main cause of aggressive and metastatic behavior of hormone-independent PCa cells^6^. Though normal prostate epithelial cells express ANXA2 abundantly, it is not oncogenic, but in PCa, the constitutively active Src phosphorylates ANXA2's tyrosine-23 residue, which is critical for ANXA2 membrane translocation and all cancer-related functions such as plasminogen activation, actin-cytoskeletal rearrangement, cellular migration, adhesion, and proliferation^7–13^. The loss of ANXA2 during PIN and early PCa, and its re-emergence in metastatic PCa is the most widely discussed protein signature^14–16^. In addition, this increase in ANXA2 expression is linked to a worse clinical outcome and cancer recurrence in patients with metastatic PCa^6^. Furthermore, earlier studies have suggested that ANXA2 may also play a role in EGFR-mediated downstream signaling^17,18^. In this regard, our previous research established the ANXA2-EGFR autocrine loop by demonstrating that ANXA2 in the cancer cell membrane plays a vital regulatory role in EGFR downstream signaling^19^. In metastatic cancer cells, downregulation of ANXA2 completely blocks all the EGFR downstream signaling, associated oncogenic events, and activates apoptosis^19^. However, the molecular mechanisms underlying the absence of ANXA2 in early PCa and its recurrence in advanced stage are yet unknown.

An altered transcriptional signature is both a cause and an outcome of cancer's hallmarks that include persistent proliferation, replicative immortality, evasion of growth suppression and apoptotic signals, angiogenesis, invasion, metastasis, evasion of immune destruction, and metabolic re-wiring^20^. The post-transcriptional processes play a significant role in determining this signature, as demonstrated by the fact that alternative RNA splicing occurs in more than half of human genes, and more than 60% of protein-coding genes contain at least one conserved miRNA-binding site^21^. However, only a few studies have looked at the involvement of specific miRNAs and their targets in the development and progression of metastatic PCa^22–26^. In this context, we investigated miRNA-mediated transcript regulations and identified that ANXA2-codon and -3′UTR-targeting miR-936 has inverse regulatory roles in the progression of androgen-independent metastatic PCa. In this study, siRNA-mediated downregulation of miRNA maturation enzymes Drosha and Dicer in hormone-dependent PCa cells (LNCap), resulted the massive induction of ANXA2 expression, and also showed that miR-936 antagomir treatment induced ANXA2 protein expression. In addition, overexpression of miR-936 destabilises the mature ANXA2 mRNA post-transcriptionally, resulting in ANXA2 near extinction in early-neoplastic PCa. However, the overexpression of miR-936 in metastatic PCa cells downregulated ANXA2 expression, related downstream activators, and oncogenic functions. Overall, the loss of ANXA2 is caused by increased expression of miR-936 during early hormone-dependent PCa, and the reciprocal regulation causes it to reappear when the miRNA level is low during metastatic PCa. Moreover, our clinical specimen observations show that miR-936 is involved in a variety of biological processes that help to limit tumour growth at the molecular level. The findings of the study could lead to the development of a novel biomarker for predicting PCa prognosis and a potential therapeutic target for improving PCa treatment.

## Materials and methods

### Cell culture and transfection

The PCa cell lines LNCaP, DU145 and PC-3 were purchased from NCCS Pune (INDIA) and cultured in RPMI-1640 (HiMedia, India) supplemented with 10% fetal bovine serum (FBS), 100 U/mL penicillin, and 100 mg/mL streptomycin, incubated at 37 °C in 5% CO2 with humidified atmosphere. Polyethylenimine (PEI 25000, Polysciences, USA) was used as a transfection reagent, and cells were seeded a day before transfection. Next day, transfection mixture was made in 150 mM NaCl solution by mixing DNA and polyethylenimine in a 1:2.4 ratio [DNA (μg): polyethylenimine (μg)] and the mixtures were incubated at room temperature for 15 min before being added drop-by-drop to the culture medium. MicroRNA expression vector, pCMV-MIR (M1005758 (#SC400690), Origene technology Inc, USA) was used to construct hsa-miR-936 plasmid vector for transfection to produce stable PC-3 cell lines using G-418 selection, subsequently named as miR-PC-3 cells. Synthetic oligonucleotide against hsa-miR-936 (HmiR-AN0841-SN-10, GeneCopoeia, USA) was used to transfect LNCaP cells for neutralizing the endogenous hsa-miR-936.

### Immunohistochemistry

Paraffin-embedded PCa tissue sections were obtained from the SDM College of Medical Sciences and Hospital, Dharwad in accordance with established core protocols and Institutional Ethical Board approval. ANXA2 protein expression in tissues collected from PCa patients, including hormone-dependent, metastatic malignant tissue and adjacent non-malignant epithelium, and also in mice tissues from animal experiments, was determined by immuno-histochemical staining as described previously^19^. First, tissue sections were stained with hematoxylin–eosin to assess the histological form and grade of tumors and then subjected to immunohistochemistry. In brief, following deparaffinization and endogenous peroxidase blockage, the sections were heated in 0.01 M citrate buffer solution (pH 6.0) in a water bath at 98 °C for 20 min; then incubated with 1:100 diluted monoclonal anti-body to ANXA2 (sc-28385, Santa Cruz Biotechnology, TX, USA) overnight at 4 °C; and visualized using a 3,3′-diaminobenzidine detection kit (Vector labs). Staining intensity of ANXA2 was graded by microscopic observation on a scale of 0 to 3+ , where 0 and 3+ indicates no staining and strong staining, respectively.

### MiRNA microarray

As described earlier^27^, the total RNA was isolated from the DU-145, PWR-1E and LNCaP cells and enriched for small RNA (< 300 nt) by size-fractionation using a YM-100 Microcon centrifugal filter (Millipore, Billerica, MA), according to the manufacturer’s instructions. The small RNAs isolated was 3′ extended with poly(A) tail using poly(A) polymerase and an oligonucleotide tag was ligated to the poly(A) tail for later fluorescent dye staining. For hybridization, the probe on the plate consisted of sequences complementary to the miRNA from miRBase and the custom sequences; the target was the RNA from the samples. After hybridization, detection used fluorescence labeling using tag-specific Cy3 dye. Images collected were analyzed using Array-Pro image analysis software. The data analysis involved subtraction of the background along with normalization using a LOWESS filter. The miRNA that was differentially expressed in the cells was identified and "BLAST"ed against the unspliced mRNA/genomic sequence of the ANXA2. The putative miRNA for the study met all of the following criteria: it was differentially expressed in the miRNA microarray analysis, had good matches with the target sequence in the BLAST search, and was predicted by one of the in-silico miRNA prediction methods.

### RNA extraction and quantitative reverse transcription-polymerase chain reaction

Total RNA from the cells was isolated using TRIzol reagent method (Thermo Fisher Scientific Inc., USA) and 1 μg of RNA was used for cDNA synthesis (Thermo Fisher Scientific Inc., USA) according to the manufacturer instructions. As described previously^19^, the quantitative reverse transcription-polymerase chain reaction (qRT-PCR) was performed using the gene-specific primers for ANXA2 (F-5′TAACTTTGATGCTGAGCGGG3′ ; R- 5′TAATTTCCTGCAGCTCCTG3′), GAPDH (F-5′GAGCGAGATCCCTCCAAA3′ ; R- 5′ACTGTGGTCATGAGTCCTT3′), miR-936 (F-5′ CAGACAGTAGAGGGAGGAATC3′ ; R- 5′GTCCAGTTTTTTTTTTTTTTTCTGC3′) and U6 snRNA (F-5′CTTCGGCAGCACATATACTAAAA3′ ; R-5′CGCTTCACGAATTTGCGTGTCAT3′) designed using Primer3 software, were used for the amplification using TB Green Premix Ex Taq II (#RR820A, Takara, Japan) in Quant Studio QS5 (Thermo Fisher Scientific Inc., USA). The relative transcripts expression level was calculated on the basis of 2^−ΔΔCT^ method, where GAPDH and U6 snRNA expression were used as endogenous control, and each gene expression was reported as n-fold change.

### Western blot analysis

Cellular proteins were extracted, quantified, separated on 10% Bis–Tris PAGE, and western blotting was performed as described previously^19^. Proteins were detected using the specific monoclonal antibodies against ANXA2 (sc-28385), pEGFR (sc-81488), pAKT (sc-7985-R), AKT (sc-5298), pSTAT-3 (sc-7993), STAT-3 (sc-8019), pERK (sc-7383), p27-kip (sc-528), GAPDH (sc-25778), HIF-1-alpha (sc-13515), VEGF (sc-7269), Vimentin (sc-32322), E-Cadherin (sc-7870), MMP-9 (sc-12759), Bcl-2 (sc-492) and Bcl-xl (sc-8392) (Santa Cruz Biotechnology, SantaCruz, CA). Appropriate Secondary antibodies conjugated to horseradish peroxidase (BioRad, USA) were incubated for 2 h at room temperature with the respective membranes. The images were acquired using enhanced Chemiluminescence system (G: BOX Chemi XX9) and the protein bands were quantified by densitometry analysis using Image J (IJ 1.46 r).

### Dual luciferase reporter assay

Dual-Luciferase assay was performed as described previously^28^. Briefly, the luciferase expression vector pGL4-human ANXA2 encoding the 3′UTR and coding region containing predicted miR-936 binding site 5′-UGUACUGU-3′ and 5′-UCUACUGU-3′, respectively, was constructed. Concomitantly, the site directed mutagenesis of miR-936 binding sites was performed to produce mutant constructs. The ANXA2 UTR-specific (infusion_1: ATC CTT TAT TAA GCT TAG CCC GAC ACG GCC TGA GCG T; infusion_2: TTA AAC AGT TAA GCT TCA TTT AAA TTT AAC TTA AAT AGC GAC AC) and ANXA2 coding region- specific (infusion_1: ATC CTT TAT TAA GCT TTC TAC TGT TCA CGA AAT CCT G; infusion_2: TTA AAC AGT TAA GCT TGT CAT AGA GAT CCC GAG CAT C) primers were used. PC-3 and LNCaP cells were transiently transfected with either WT or mutated reporter constructs and pre-miR-936 using polyethylenimine in a 24-well plate, and co-transfected with anti-miR-936 after 48 h of transfection, then the cells were harvested and lysed for the assay. Luciferase activity was measured according to the Dual-Luciferase Reporter Assay System (Promega, USA), and renilla luciferase expression was used for data normalization.

### Wound healing assay

In vitro scratch assay was performed as described previously^19^. Briefly, 6 × 10^5^ PC-3 and stable miR-936 expressing PC-3 (miR-PC-3) cells were seeded onto six-well plate, after reaching confluency, a scratch was made using 200 μL pipette tip. The open space was tracked using live cell imaging microscope (Motic AE2000 series camera), photographed at 0, 6, and 18 h interval, and the percentage of wound closure was quantified using Image J (IJ 1.46r).

### Colony formation assay

Five thousand PC-3 and/or miR-PC-3 cells were seeded in 35-mm culture dish and allowed to grow for 12–15 days to form the colonies. Surviving cell colonies were fixed with 3.7% formaldehyde, washed with PBS, and stained with 0.2% crystal violet solution and 2% ethanol. Plates were photographed, and the percentage of surviving colonies was quantified using Image J (IJ 1.46 r).

### Xenograft tumor assay

As previously described, in vivo tumorigenicity studies were conducted in compliance with the Institutional Animal Ethics Committee (IAEC) of SDM College of Medical Sciences and Hospital, Dharwad^29^. In brief, 4–5 weeks old, 16–19 g male BALB/c nude mice were used for in vivo tumorigenicity studies. The suspensions of PC-3 and miR-PC-3 cells (~ 1 × 10^7^ cells/mL) were mixed with Matrigel and injected (n = 4 per group) subcutaneously into the flank of mice. The tumor size was observed regularly up to the onset of palpable tumor and all the mice were injected intraperitoneally with ketamine (90 mg/kg) to anaesthetize and euthanised by cervical dislocation to collect the tumor. Visual images of the tumor were immediately photographed after the excision. To measure the length (L) and width (W) of tumors using digital Vernier Caliper, and the tumor size was calculated using the following formula: V = ab^2^/2 (a, length; b, width).

### Ethics approval and consent participate

The study was conducted in accordance with the Declaration of Helsinki, and approved by the Institutional Ethics Committee of SDM College of Medical Sciences and Hospital (SDM-IEC-54/Ext-2016, and 08-07-2016) for studies involving humans. Informed consent was obtained from all subjects involved in the study. The animal study protocol was approved by the Institutional Animal Ethics Committee of SDM College of Medical Sciences and Hospital (SDM-IAEC-53/Ext-2016, and 08-07-2016) for studies involving animals and confirming that all experiments were performed in accordance with the ARRIVE guidelines as stipulated in the Indian Committee for the Purpose of Control and Supervision of Experiments on Animals (CPCSEA), a statutory Committee of Department of Animal Husbandry and Dairying (DAHD), Ministry of Fisheries, Animal Husbandry and Dairying (MoFAH&D) constituted under the Prevention of Cruelty to Animals (PCA) Act, 1960 regulations.

## Results

### Altered expression of ANXA2 in prostate cancer

**Figure 1:**
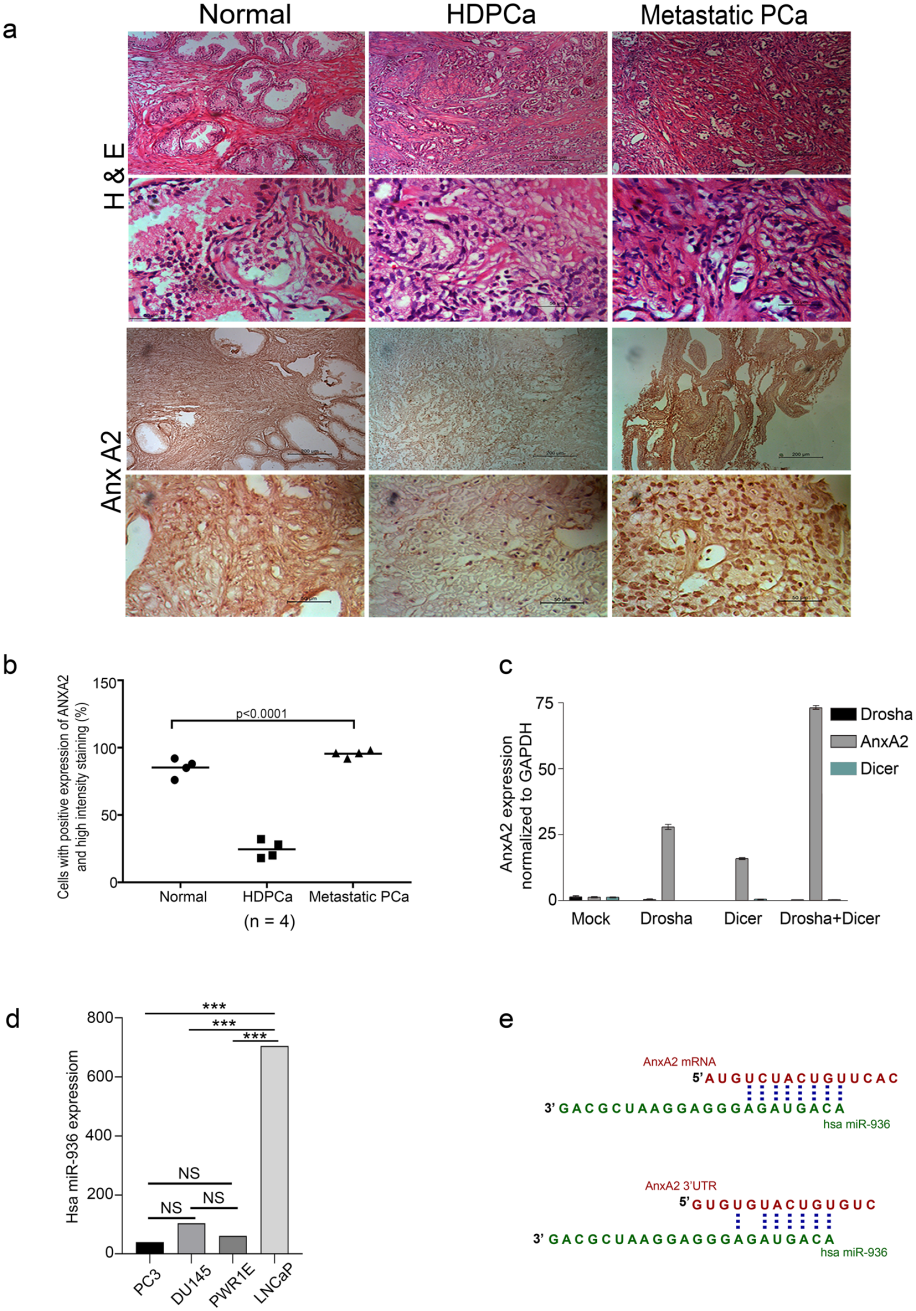
Representative Immunohistochemical (**a**) and its analytical data (**b**) demonstrating ANXA2 expression pattern in clinical PCa specimens. (**c**) qRT-PCR analysis shows the knockdown of miRNA maturation enzymes Drosha and Dicer in LNCap cells induces the ANXA2 overexpression. (**d**) Microarray profiling identified significant expression of hsa-miR-936 in LNCaP cells, compared to PC-3, DU145 and PWR1E (***p < 0.0001). (**e**) miR-936 binding to codon and 3′UTR region of ANXA2 mRNA.

To confirm the altered expression of ANXA2 in PCa, its expression level in different stages of PCa was determined by immunohistochemical analysis. As observed in earlier studies, our immunohistochemistry data also demonstrated the significantly (P < 0.0001) high expression of ANXA2 in normal prostate epithelial cells with 2+ staining intensity. In PIN and during early PCa incidence, the ANXA2 is very low or null with 0 or 1+ staining intensity. However, the higher Gleason score and poorly differentiated PCa specimen demonstrated abundant expression of ANXA2 with 3 + staining intensity (Fig. 1a,b).

### Downregulation of miRNA biogenesis components induces ANXA2 expression in hormone-dependent prostate cancer

The siRNA mediated downregulation of miRNA maturation enzymes Drosha and Dicer in hormone-dependent PCa LNCaP cells resulted in massive induction of ANXA2 expression. Drosha siRNA treatment alone induced nearly 30-fold increase in ANXA2 mRNA level, Dicer siRNA treatment alone increased about 16-fold and both together increased 70-fold ANXA2 mRNA expression (Fig. 1c). These results clearly indicate that disappearance of ANXA2 in LNCaP cells is due to miRNA mediated destabilization of ANXA2 mRNA.

### MicroRNA profiling identified the differential expression of miR-936 in hormone-dependent and -independent prostate cancer

MicroRNA profiling of normal prostatic epithelial cells PWR1E, early hormone-dependent PCa cells LNCaP and metastatic PCa cells DU145 and PC-3 identified hsa-miR-936 as the most differentially expressed miRNA. Further in silico and expression analysis revealed that miR-936 expression was minimal in PC-3, PWR1E and DU145 cells, and was very highly expressed in LNCaP cells (Fig. 1d). Moreover, the increased expression of miR-936 resulting in near absence of ANXA2 was observed in early neoplastic stage as this miR-936 has a seed sequence complimentary to the ANXA2 coding region that post-transcriptionally destabilizes the synthesis of mature mRNA (Figs. 1a,b,e). Furthermore, another complimentary seed sequence in the 3’UTR region of ANXA2 was also identified for miR-936 (Fig. 1e).

### MiR-936 antagomir treatment brings back ANXA2 expression in hormone-dependent prostate cancer

**Figure 2:**
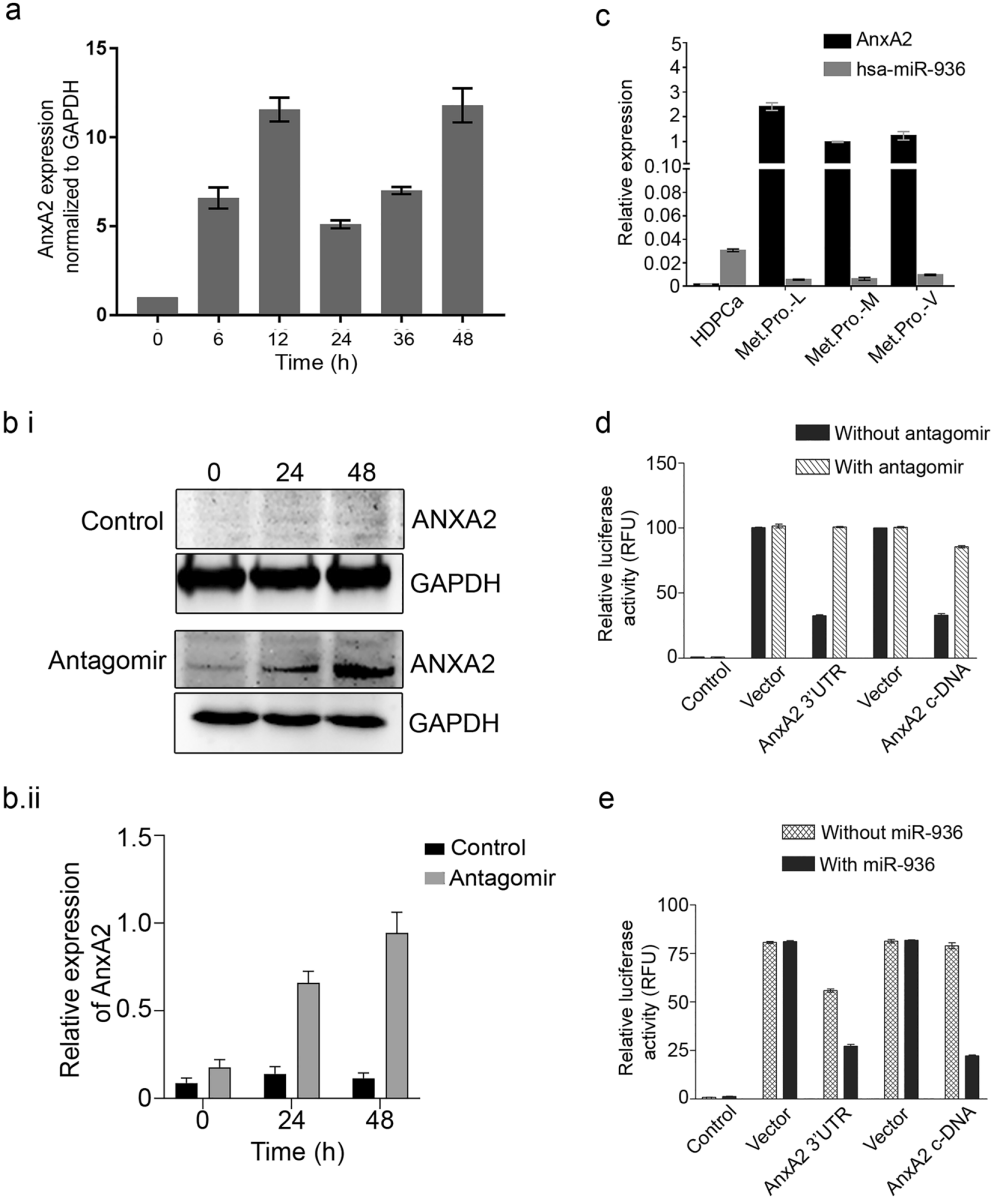
MiR-936 antagomir treatment to ANXA2-null LNCaP cells induces ANXA2 expression at mRNA (**a**) and protein (**b**) level. qRT-PCR analysis showing the reciprocal regulation of miR-936 and ANXA2 in hormone-dependent (HDPCa, Male, Age 71 years, Gleason Score (GS-6) and metastatic PCa samples (Met.Pro-L, Male, Age 72 years, GS-9; Met.Pro-M, Male, Age 85 years, GS-8; Met.Pro-V, Male, Age 70 years, GS-9) (**c**). Luciferase Reporter Assay showing relative luminescence upon co-transfection of miR-936 antagomir with either empty luciferase vector or ANXA2 cDNA/3′UTR luciferase vector (ANXA2WT/ANXA2Mut) in LNCaP cells (**d**), co transfection with miR-936 in PC-3 (**e**) respectively. Both the values are highly significant, P ≤ 0.001.

Considering the null expression of ANXA2 and relatively very high expression of miR-936, an androgen dependent PCa cell line LNCaP was selected for the antagomir treatment experiment. ANXA2 mRNA expression was induced within 6 h of miR-936 antagomir treatment by qRT-PCR and observed stable till 48 h post treatment (Fig. 2a). Additionally, the reappearance of ANXA2 protein expression was observed by western blot analysis in ANXA2-null LNCaP cells after 48 h of miR-936 antagomir treatment, demonstrates the role of miR-936 in the destabilization of ANXA2 mRNA during early PCa (Fig. 2b).

### MiR-936 downregulation is a key driver of the transition from hormone-dependent to hormone-independent metastatic prostate cancer

In order to clarify the involvement of miR-936 in the transition of hormone-dependent to hormone-independent metastatic PCa, tissue samples obtained from PCa patients were analysed for miR-936 and ANXA2 expression by qRT-PCR. The expression of miR-936 was significantly decreased in metastatic PCa compare to hormone dependent PCa, where miR-936 expression was significantly increased. On the contrary, ANXA2 mRNA level is negligible in Hormone dependent PCa compared to very high expression in metastatic PCa tissue samples (Fig. 2c). This clinical data convincingly illustrates the involvement of miR-936 in driving the very high expression of ANXA2 in hormone-independent metastatic PCa.

To ascertain the clinical sample observations, a dual-luciferase reporter assay was performed to confirm the presence of direct interaction between miR-936 and ANXA2 mRNA. An infusion cloning method was adopted to generate a reporter plasmid that is driven by the cytomegalovirus basal promoter and harbouring the ANXA2 coding and 3’UTR nucleotides at the 3′ position of the Luciferase reporter gene. The transient transfection of ANXA2 reporter constructs expressing plasmid into LNCaP cells where basal miR-936 expression is high, reduced the luciferase activity of plasmid but not affected by co-transfection with miR-936 antagomir (Fig. 2d). However, the luciferase activity of plasmid was not affected in PC-3 cells where miR-936 expression is expected to be very low, but significantly decreased after co-transfection with miR-936 expressing plasmid (Fig. 2e). These results indicated that miR-936 interacted with the specific coding region and 3’UTR of ANXA2 mRNA.

### MiR-936 regulates ANXA2 expression and its mediated ANXA2-EGFR signaling axis

**Figure 3:**
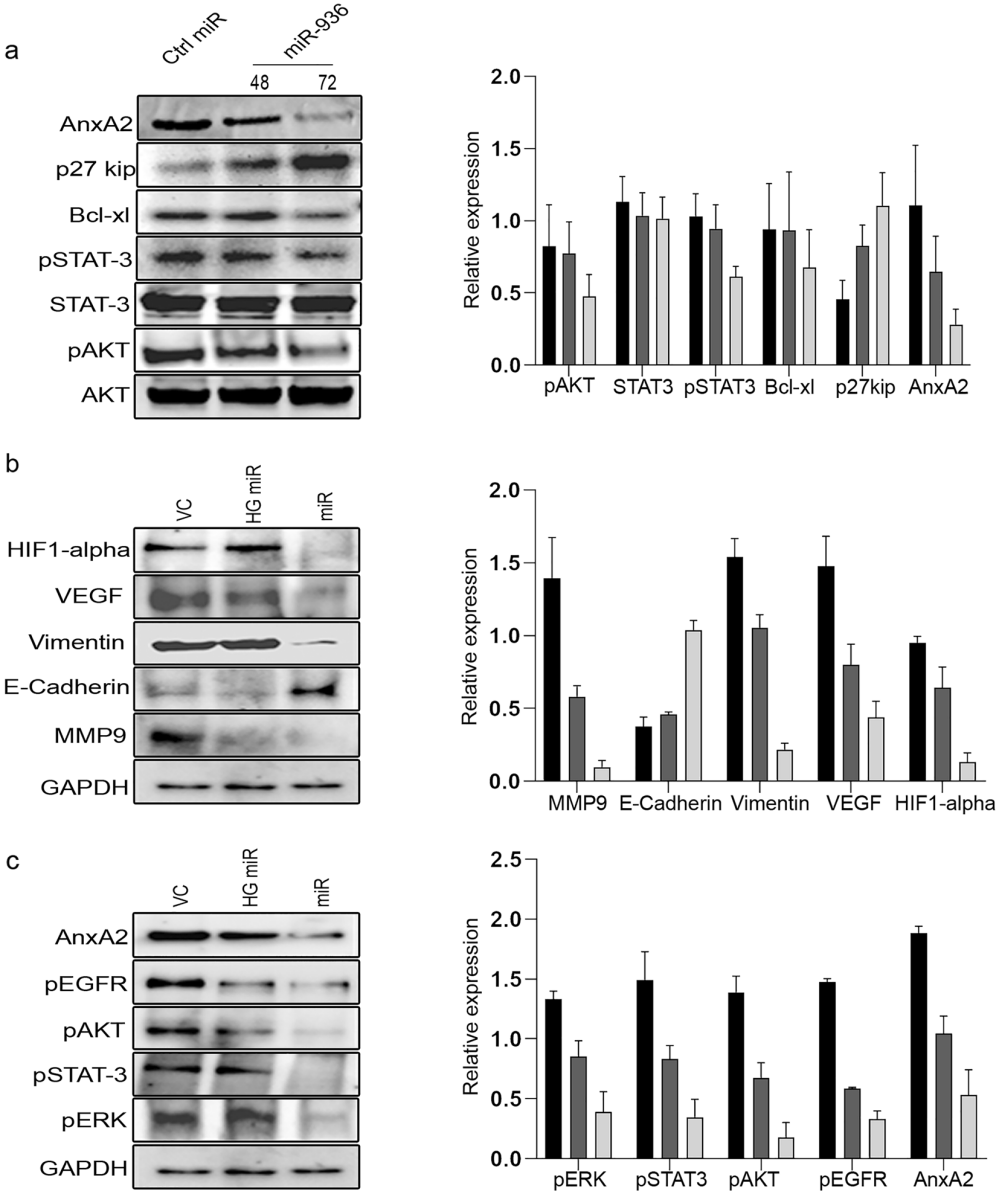
PC-3 cells ectopically expressing the vector control or miR-936 for 48 h were immunoblotted for the respective proteins, and AKT was used as a loading control (**a**). Western blot data showing the expression of downstream signaling (**b**) and functional targets (**c**) of ANXA2 upon stable transfection of Pre-miR-936 in PC-3 cells and GAPDH was used as a loading control. To correlate the expression pattern, Vector control (VC) was compared with Heterogenous stable cells (HG-miR) and single cell clone of PC-3 expressing miR-936 (miR).

The loss of miR-936 in PC-3 cells and metastatic PCa was demonstrated to possess the higher tumorigenicity mediated by the expression of ANXA2, was further assessed for its effect on ANXA2-EGFR mediated downstream signaling. Transient transfection of miR-936 in PC-3 cells inhibited ANXA2 protein expression after 72 h of treatment. Consistent with the decrease of ANXA2 expression, anti-apoptotic Bcl-xl and signaling molecules like pAKT and pSTAT-3 expressions were also decreased at the protein level following the transient transfection of miR-936. Conversely p27kip, a putative tumor suppressor expression was increased, while the expression of STAT-3 and AKT remain unchanged (Fig. 3a).

The ANXA2 mRNA and protein expression in PC-3 cells was significantly knockdown with the transfection of miR-936 vector by gain-of-function assay. In accordance with the reduction in ANXA2 expression, the ectopic expression of miR-936 had the same effect on downstream effectors of ANXA2 such as VEGF, HIF-1alpha, vimentin, and MMP-9, however increase in E-Cadherin was also observed (Fig. 3b). Similarly, pEGFR and its other downstream signaling molecules like pAKT, pSTAT-3 and pERK expressions were also downregulated at the protein level following overexpression of miR-936 (Fig. 3c).

### MiR-936 overexpression attenuates PCa cell growth and metastasis

**Figure 4:**
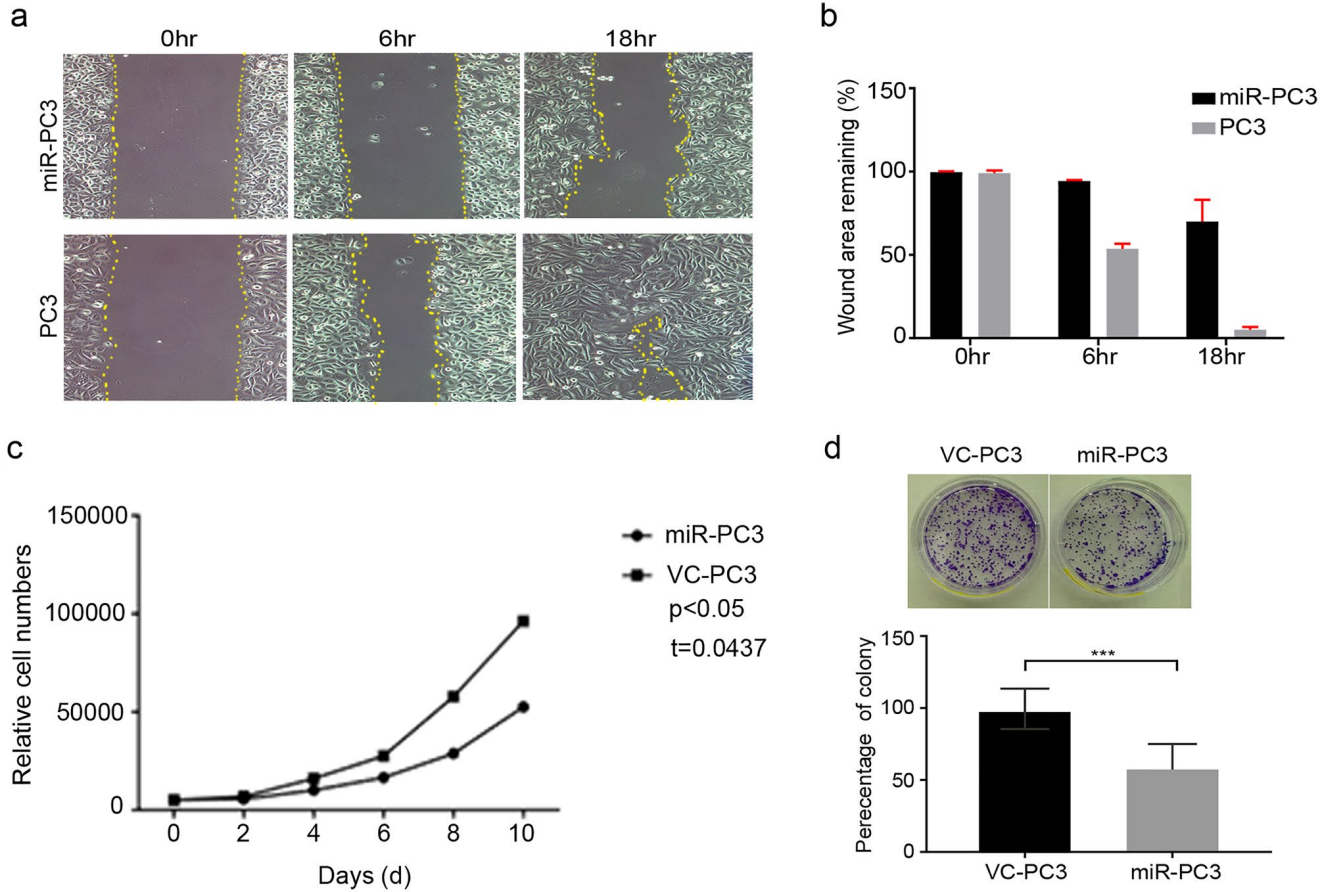
Overexpression of miR-936 inhibits PCa cell growth, migration and colony formation. (**a**) Vector control PC-3 and miR-PC-3 cells were grown in six well plate, once cells become confluent, wounds were made and the (**b**) relative ratio of wound closure per field during different time duration is shown. (**c**) Growth of PC-3 cells were compared with stable PC-3 cells expressing miR-936, the growth index was assessed at 2, 4, 6, 8 and 10 days. (**d**) Crystal violet assay showing number of colonies formed by vector control PC-3 cells or pre-miR-936 expressing stable PC-3cells after 12 days on tissue culture treated adherent plates.

The results of cellular function assays demonstrated that over expression of miR-936 in PC-3 cells had markedly decreased the proliferation, colony formation, migration, and invasion processes of the metastatic PCa PC-3 cells. Given that ANXA2 has a critical role in the regulation of cell proliferation, colony formation and migration, metastatic PCa cells, PC-3 were transfected with miR-936 and analyzed for these terminal functions. The migration potential of miRNA-936 expressing PC-3 cells significantly abrogated compared to wild type PC-3 cells (Fig. 4a,b). Cell proliferation of PC-3 cells were compared with stable PC-3 cells expressing miR-936 (miR-PC-3). The proliferation index was assessed at 2, 4, 6, 8 and 10 d (Fig. 4c) till ten days the rate was high in PC-3 compared to miR-PC-3 cells, after ten days significant 50% inhibition in proliferation was noticed in miR-PC-3 cells. Colony formation assay revealed that miRNA overexpression in PC-3 cells leads to significant decrease in the number as well as size of colonies compared to vector control PC-3 wild type cells as reported in Fig. 4d. Results taken together suggest that cancer cell proliferation, colony formation and migratory potential is inhibited by miR-936 predominantly through downregulation of ANXA2-EGFR signaling and inactivation of terminal functional proteins.

### MiR-936 inhibits subcutaneous tumor formation in vivo

**Figure 5:**
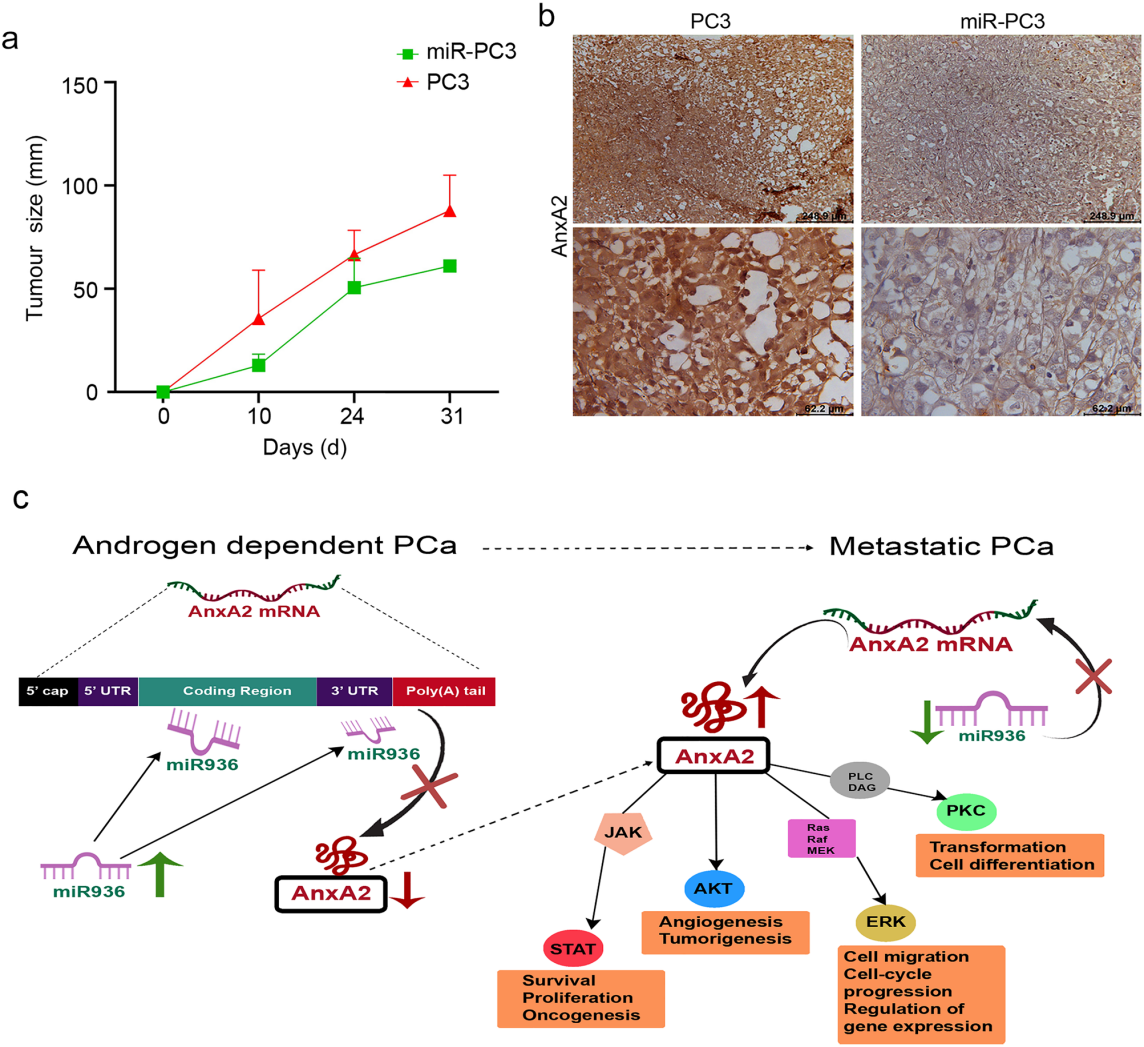
MiR-936 overexpressing PC-3 stable cell line expresses very low expression of ANXA2 compared to basal expression of PC-3 cells. Similarly, tumorigenesis effect is nullified with miR-936 overexpressing cells in xenograft studies (**a,b**). Proposed model was prepared in Adobe^®^ Photoshop^®^ CS2 version 9.0 explains the altered expression of ANXA2 in hormone-dependent and -independent metastatic PCa. The disappearance of ANXA2 expression is associated with abundant expression of tumor suppressor miR-936 in hormone-dependent PCa. This miR-936 binds directly to both coding as well as 3′UTR region of ANXA2 mRNA and downregulate the expression of ANXA2 in hormone-dependent PCa. However, the low expression of miR-936 in metastatic hormone-independent PCa was found to be responsible for the induction of the ANXA2 expression. Further, this calcium-dependent phospholipid-binding protein ANXA2 is known to acts as an upstream activator for all the pro-survival downstream signaling molecules like JAK, STAT, AKT and ERK. Subsequently, it results in increased cell growth, survival, proliferation, oncogenesis, migration, and cell cycle progression in metastatic PCa (**c**).

We looked into the tumorigenic inhibitory potential of miR-936 in PCa progression. During examination, we made xenografts of PC-3 cells expressing either miR-936 or vector control in male BALB/c nude mice to demonstrate miR-936's growth-repressive effect on PCa in vivo. Tumor growth was measured after a palpable tumor appeared. The tumor sizes were tracked every 5 d until the 31st day (Fig. 5a). As a result, the over expression of miR-936 in PC-3 cells, the tumor showed significant decreases compared to the group treated with vector control. These findings demonstrated that ectopic expression of miR-936 significantly suppressed tumor growth and also our immunohistochemistry data reveals the reduced ANXA2 expression in xenografts of PC-3 cells ex-pressing miR-936 in male BALB/c nude mice compared to vector control (Fig. 5b).

## Discussion

PCa is the most commonly diagnosed and is the second most leading cause of cancer-related deaths among men^30^. Androgens and the androgen receptor (AR) play an important role in prostate pathobiology as they are required for their normal growth and maintenance^23,31^. The vast majority of PCa are androgen-dependent tumors. Androgen deprivation therapy is often used as an adjuvant treatment along with radiotherapy. Even though patients initially respond well to hormonal therapies, tumors will eventually progress to develop a castration-resistant PCa (CRPC), a treatment-insensitive, metastatic disease with a worse prognosis^32,33^. The majority of patients develop CRPC and progress to metastatic disease. In addition to androgens, prostate growth and function is in-part regulated by several growth factors and their cognate receptors, one of which is the epidermal growth factor and its receptor (EGFR) has been known to drive hormone independent PCa progression. EGFR alone cannot drive the progression of metastatic PCa tumours, but its linked to its upstream fibrinolytic receptor ANXA2, are together known to the PCa and breast cancer progression^19,34^. Further, our in silico analysis revealed that the survival ability of the prostate cancer patients is poor with the increased expression of ANXA2 along with the high (> 6.0) gleason score (Fig. S1). However, the molecular mechanism underlying the progression from hormone-dependent PCa to metastatic CRPC is poorly understood. Also, the primary rationale behind the absence of ANXA2 in early PCa and its reappearance in advanced PCa are not yet deciphered. Interestingly, it has been observed that ANXA2 and EGFR expression is reduced in hormone-dependent PCa but abundant in metastatic PCa, suggesting their functional role in PCa progression that the function of these molecules may depending on the cellular context^34^. Furthermore, overexpression of ANXA2 in certain tumor is correlated with worst clinical outcomes and also it has been associated with a variety of oncogenic functions, including signal transduction, cytoskeletal rearrangement, membrane fusion, cellular migration, adhesion, and proliferation^34,35^.

MicroRNAs, a class of post-transcriptional regulators were found to be active in carcinogenesis, particularly PCa. Extensive research revealed that miRNAs could influence downstream messenger RNAs (mRNAs) via complementary base pairing, therefore influencing pathway signal transmission and the function of cellular processes^36^. Comprehensive exploration of miRNAs associated with PCa development and progression will help to improve our understanding of the molecular basis of pathogenesis. As the differential expression of miRNA tends to be both the cause and the outcome of oncogenesis in cancer^37^, we looked into the possibility of miRNAs regulating ANXA2 at the posttranscriptional level in PCa. In support to this, our initial microarray analysis of androgen-dependent cell line LNCaP, metastatic PCa cell line DU145 and normal prostatic epithelial cells PWR1E, revealed that miR-936 was the most differentially expressed. It indicates that miR-936 is involved in the development of androgen-dependent PCa and its transformation into metastatic CRPC. In recent studies, this miR-936 has been shown to influence the cell activity and tumour growth of gastric cancer and Laryngeal Squamous Cell Carcinoma^38,39^. However, the precise role and mechanism underlying the involvement of miR-936 in the prostate cancer progression has not been determined yet. In the present study, we have shown that miR-936 is strongly expressed in hormone-dependent PCa cell line LNCaP that lacking ANXA2. In addition, we also found that miR-936 expression is minimal in metastatic PCa cell lines DU145 and PC-3 where ANXA2 expression is high. Furthermore, we identified that miR-936 have direct interaction with ANXA2-coding & -3UTR. By interacting with their target mRNAs, miRNAs post-transcriptionally control gene expression by regulating translational attenuation via miRNA-binding sites in the target gene's 3'UTR and these target genes are often involved in controlling crucial developmental events. However, interactions between miRNAs and other areas have been discovered, including the 5′ UTR, coding sequence, and gene promoters. According to some recent studies, miRNAs can target the coding region of mRNA and control the gene expression^40–43^.

ANXA2 at the cancer cell membrane surface interacts with EGFR and it is critical for the regulation of downstream signalling^19^. As ANXA2 has a crucial function in the pathogenesis of PCa, current findings identified a correlation between dysregulated miR-936, overexpressed ANXA2 and PCa cell proliferation. As per our findings, ANXA2 is a direct target of miR-936 and also treatment with miR-936 antagomir induced ANXA2 mRNA as well as protein expression in ANXA2 null hormone-dependent PCa cell line LNCaP. These results were in similar to the upregulation of AR expression in LNCaP cells treated with miR-185 antagomir. However, overexpression of miR-936 in metastatic PCa cells PC-3 reduced ANXA2 expression, associated downstream activators, and oncogenic functions by inhibiting their proliferation, cell cycle progression, cell migration, and invasion abilities in vitro. Correspondingly, ectopic expression of miR-940 in metastatic PCa cell line DU145 and PC-3 attenuates its migration and invasion ability by regulating migration and invasion enhancer 1 (MIEN1) expression^27^. Furthermore, xenografts of PC-3 cells expressing miR-936 in male BALB/c nude mice significantly reduced ANXA2 expression as well as subcutaneous tumor formation in vivo, indicating a tumor suppressor role of miR-936 in PCa. Overall, Fig. 5c depicts the demonstration of the near elimination of ANXA2 by overexpression of tumor suppressor miR-936 that directly targets ANXA2-codon & -3’UTR in metastatic PCa.

## Conclusion

Current study convincingly demonstrates that miR-936 is a novel post-transcription regulator of calcium dependent phospholipid binding protein ANXA2 in hormone-dependent and -independent PCa. Moreover, this potential tumor suppressor miR-936 regulates the ANXA2 mRNA expression by binding to its coding and 3’UTR regions. Nonetheless, these results would pave the way to further research for unravelling the role of miR-936 in prostate cancer, possibly as an early diagnostic and, later as a prognostic indicator for the therapeutic management of PCa.

## Supplementary Information

Supplementary Information.

Supplementary Figure S1.

## Data Availability

The authors declare that the data presented in this study are available within the manuscript and its supplementary information files.
