# Supplementary material for: Loss of miR-936 leads to acquisition of androgen-independent metastatic phenotype in prostate cancer
Source: Sci Rep. 2022 Oct 12;12:17070. doi: 10.1038/s41598-022-20777-5 (PMC9556567; doi:10.1038/s41598-022-20777-5)

# Original Blots for Figure 4

**GAPDH**

VC HG-miR miR

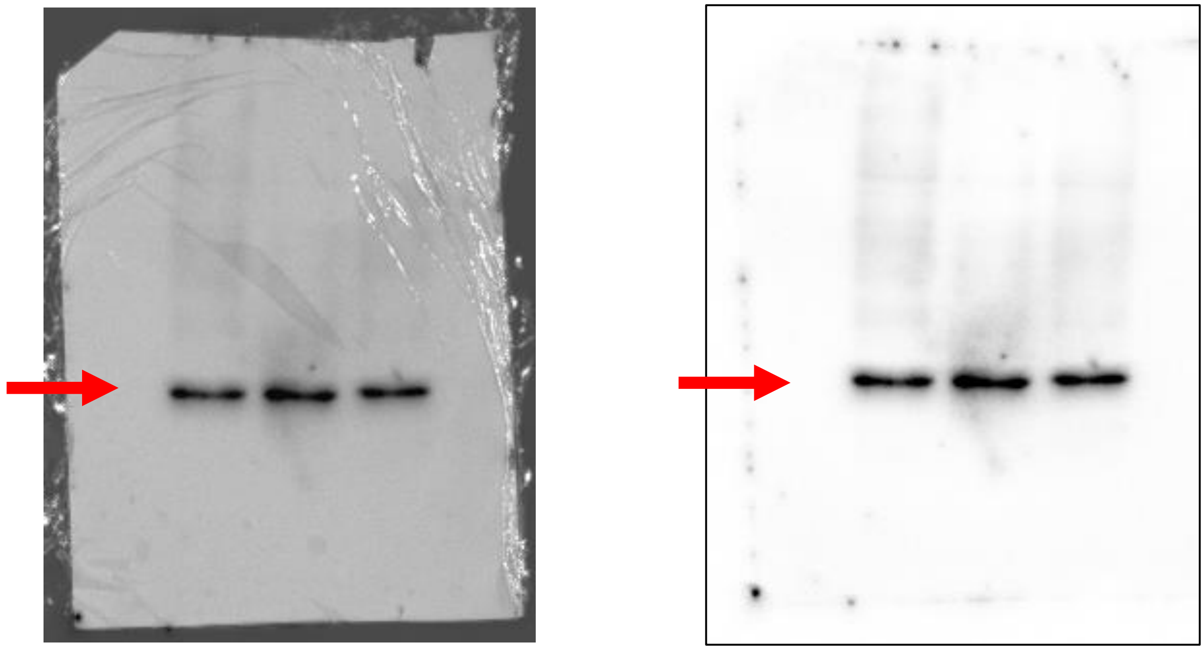

**AnxA2**

VC HG-miR miR

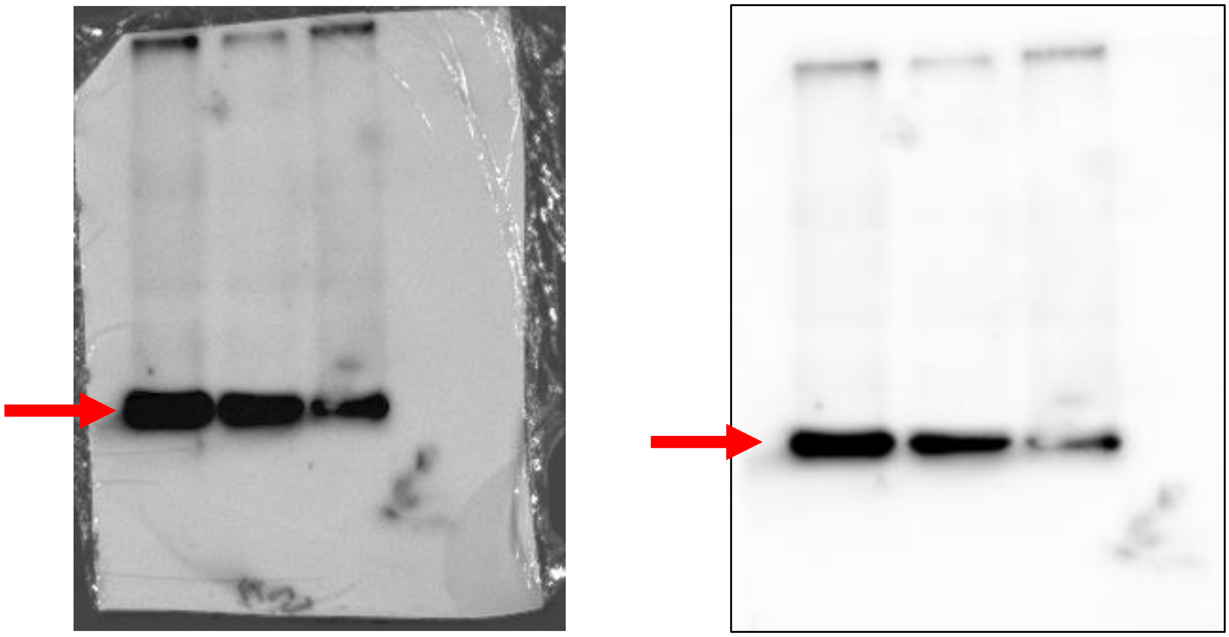

VC: Vector Control; HG miR: Heterogenous MicroRNA

## pEGFR

VC HG-miR miR

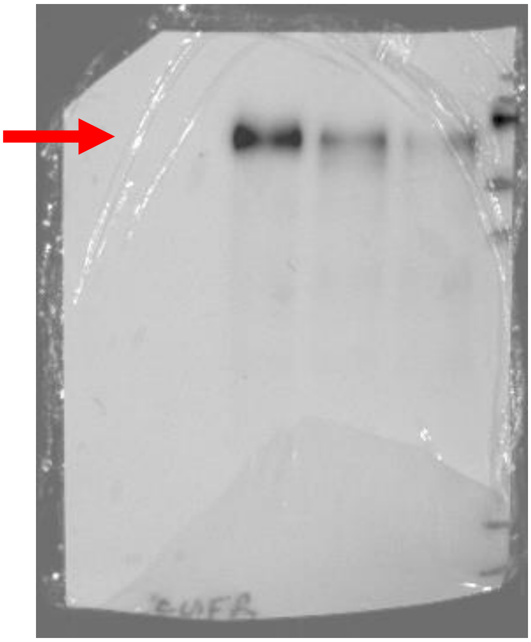

VC HG-miR miR

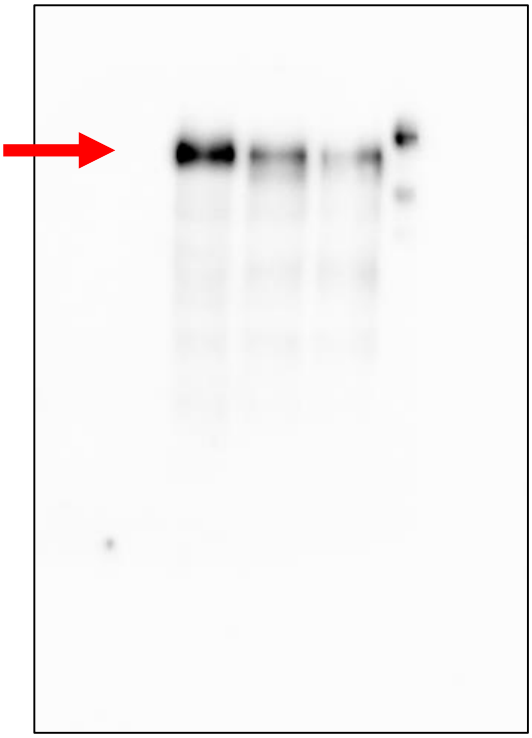

## pAKT

VC HG-miR miR

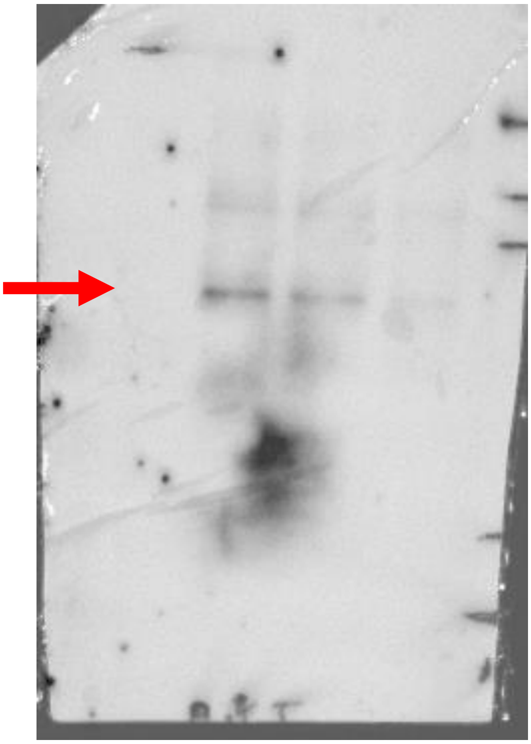

VC HG-miR miR

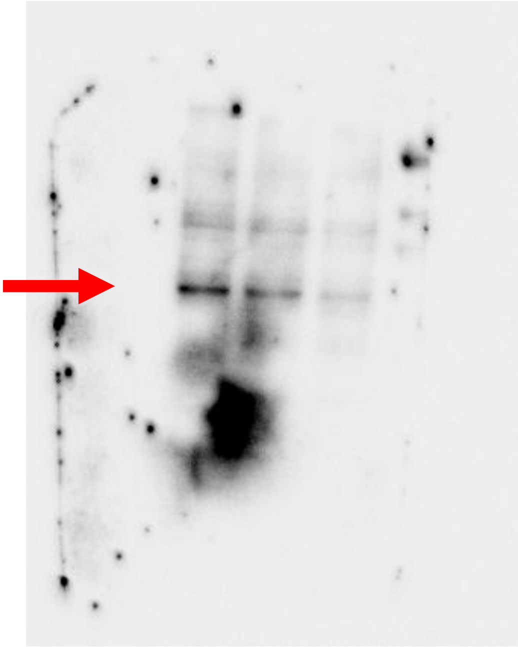

## pSTAT3

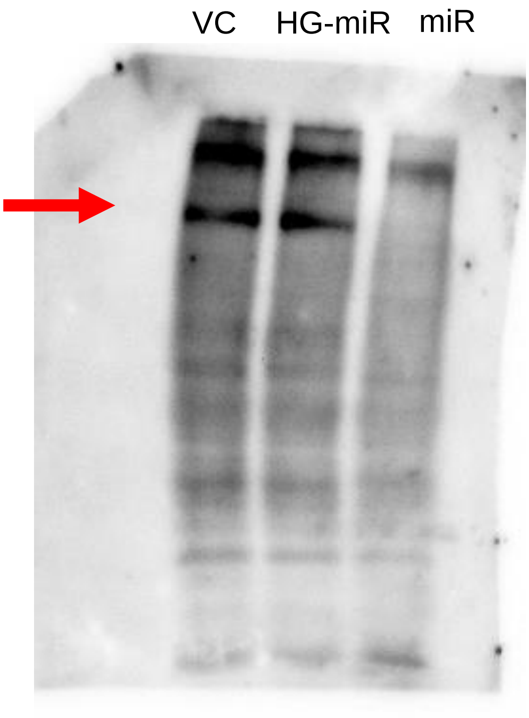


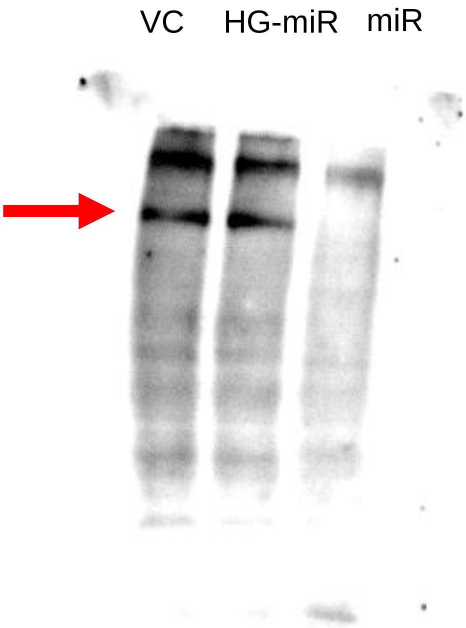


## pERK

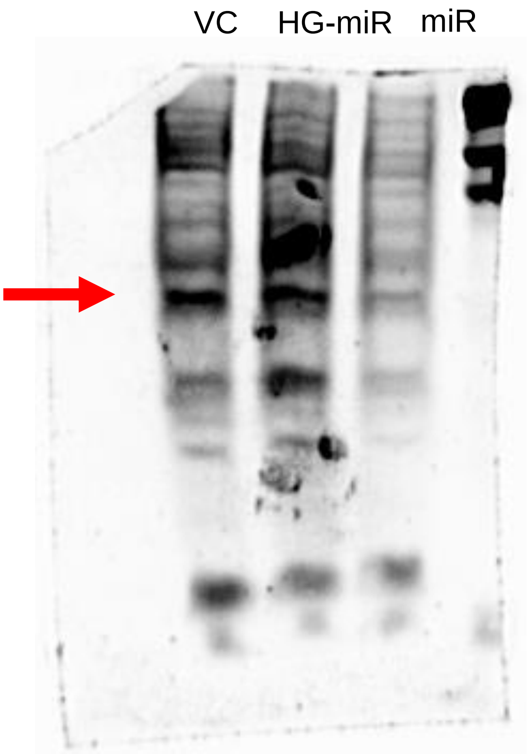


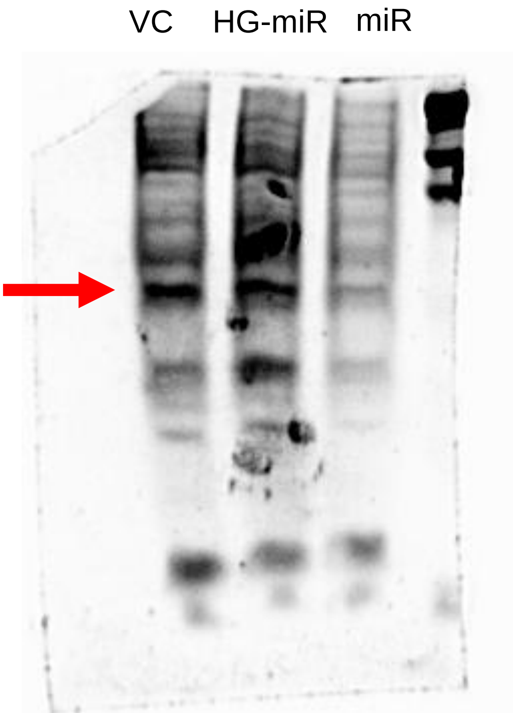

**HIF1-alpha**

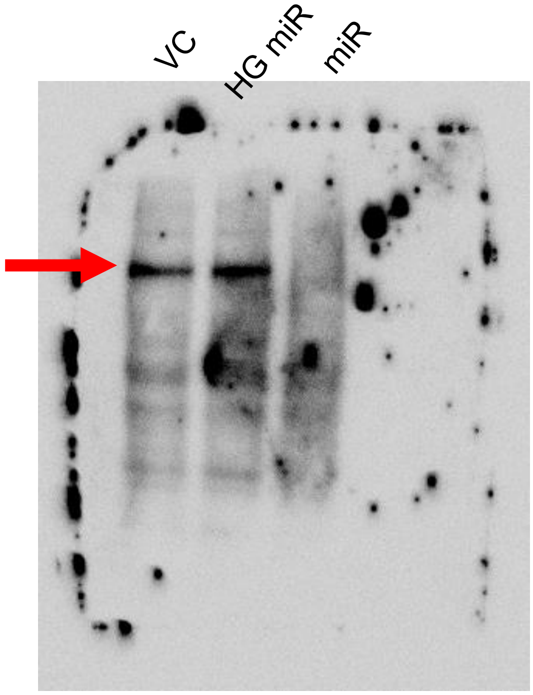


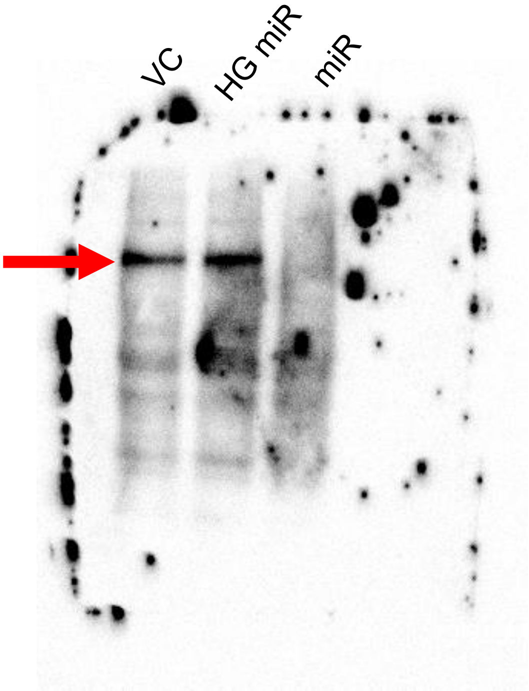


**VEGF**

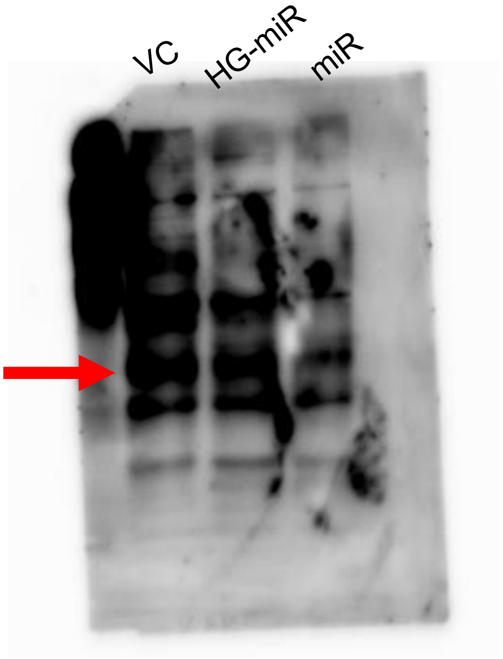


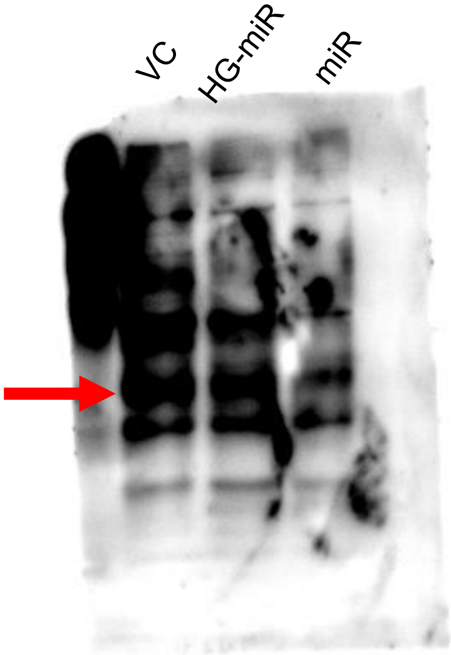

## Vimentin

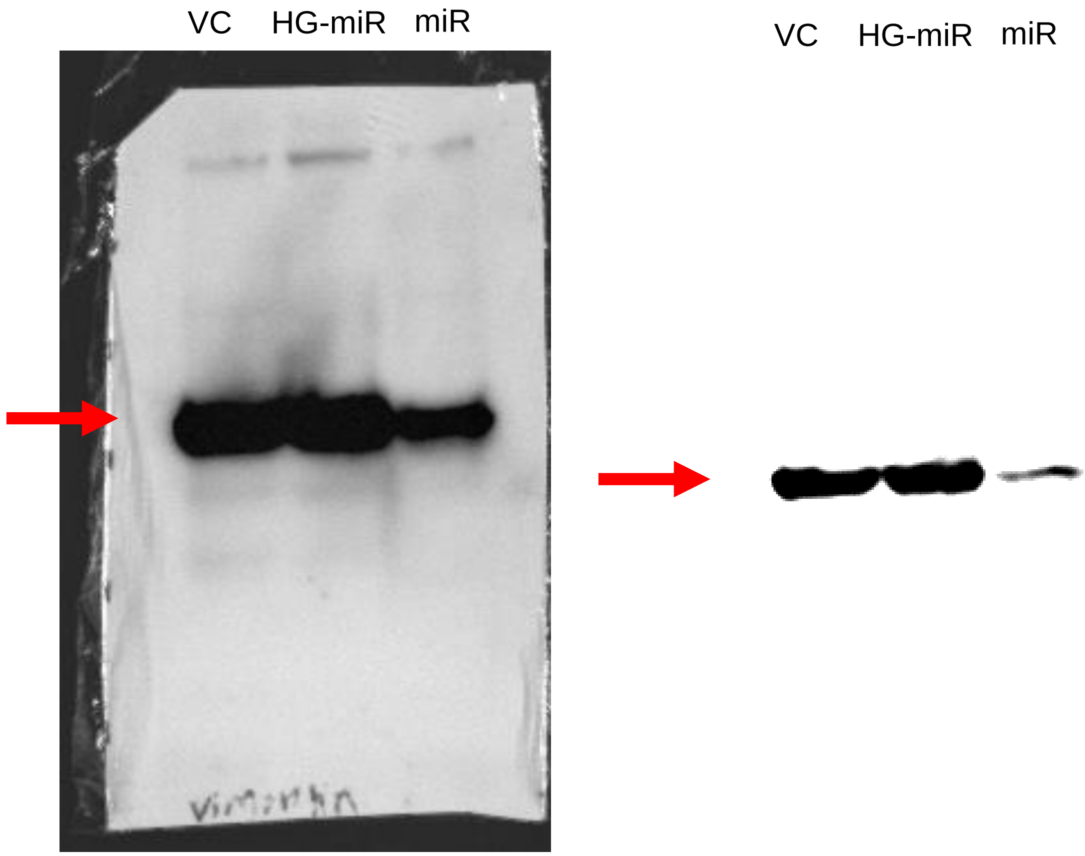


## E-cadherin

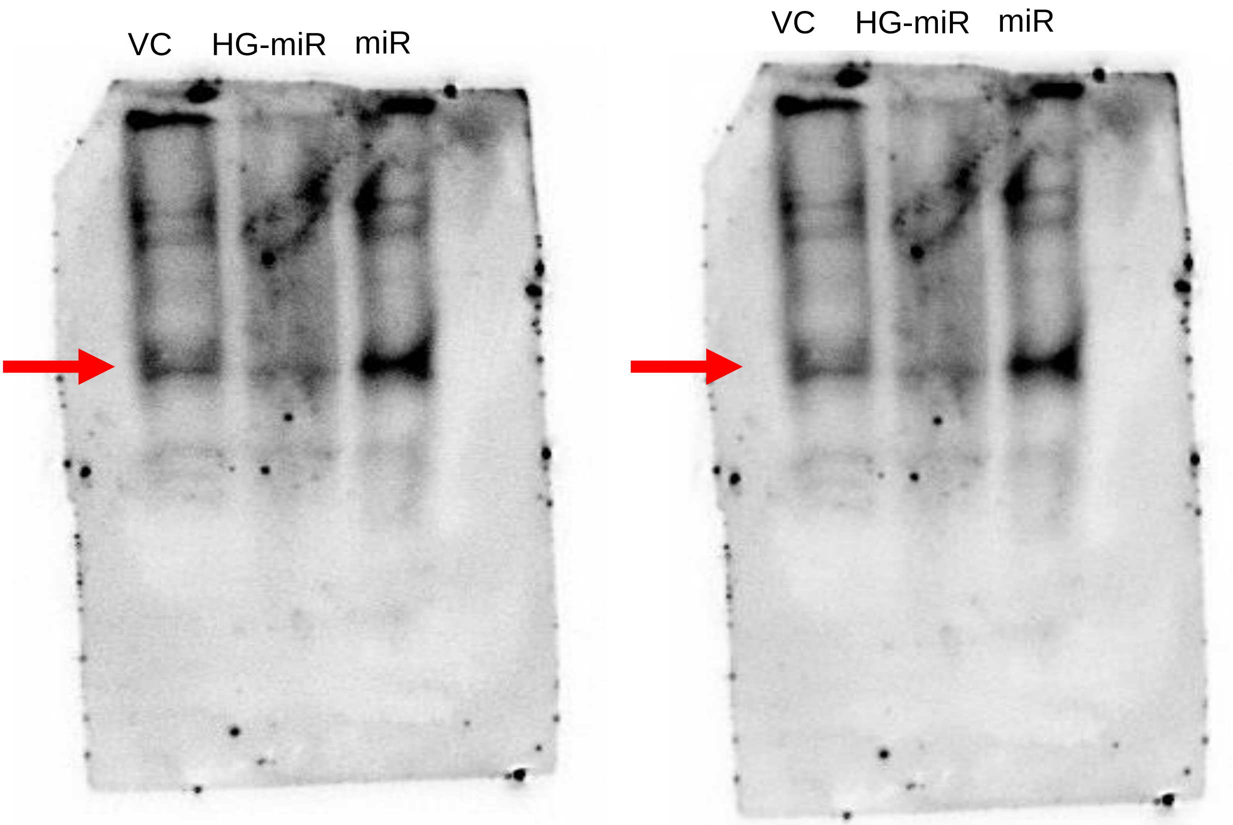

# MMP-9

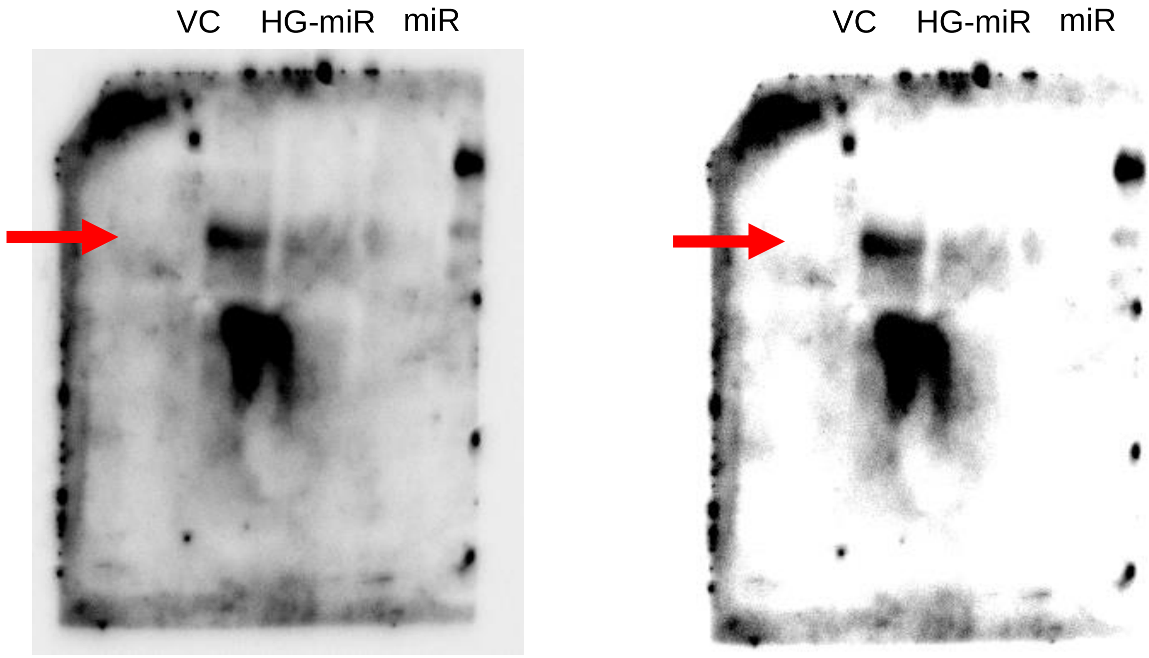

Supplement: Supplementary file 1 — Supplementary Information. [file 41598_2022_20777_MOESM1_ESM.pdf]
