# Supplementary material for: Loss of miR-936 leads to acquisition of androgen-independent metastatic phenotype in prostate cancer
Source: Sci Rep. 2022 Oct 12;12:17070. doi: 10.1038/s41598-022-20777-5 (PMC9556567; doi:10.1038/s41598-022-20777-5)
Supplement: Supplementary file 2 — Supplementary Figure S1. [file 41598_2022_20777_MOESM2_ESM.pdf]

Supplementary Information

Loss of miR-936 leads to Acquisition of Androgen-independent Metastatic Phenotype in Prostate Cancer

Sarathkumar Edachery [1,2], Prakash Patil [3], Rajashekar Mohan [4], Bhuvanesh Aradhya [5], Jayaprakash Shetty [6], Shama Prasada Kabekkodu[7], Manas Kumar Santra [8], Sathisha Jayanna Gonchigar [1,*] and Praveenkumar Shetty [3,9,*]

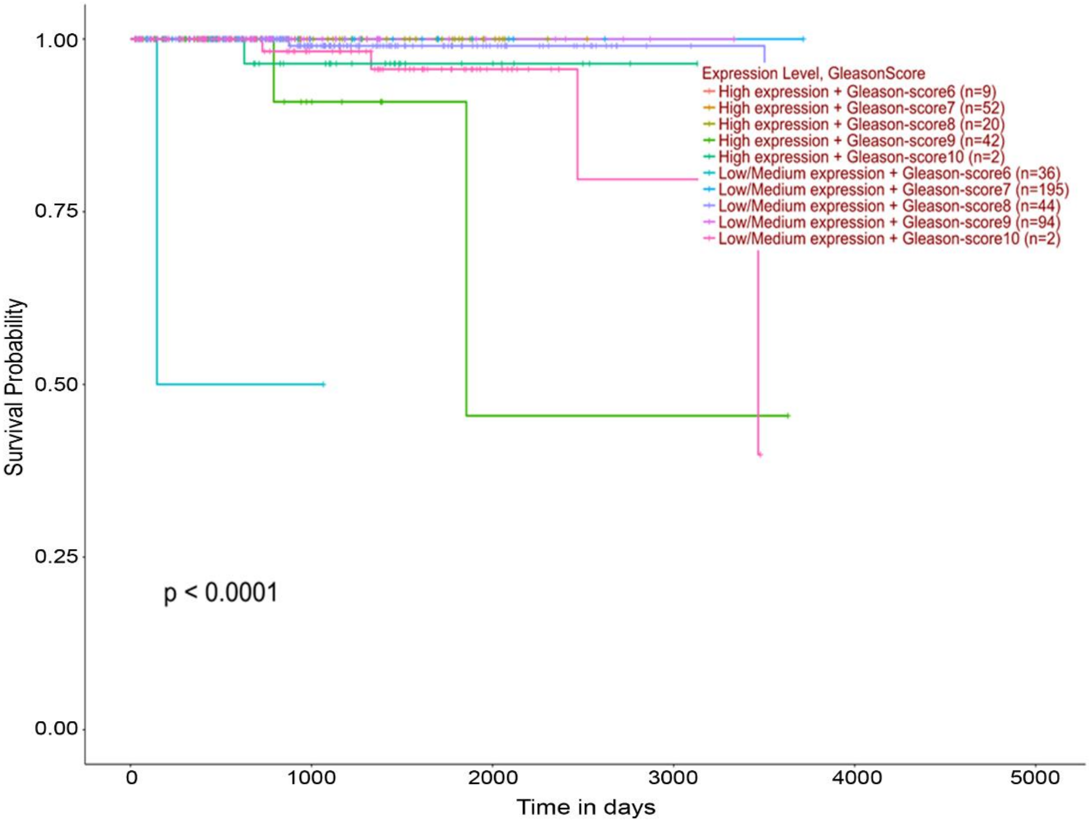

Fig. S1. The association between ANXA2 expression combined with Gleason score in predicting survival of prostate cancer patients. The impact of ANXA2 expression combined with the Gleason score in predicting the overall survival (OS) in the TCGA-PRAD cohort was analyzed using the UALCAN tool. The patient survival was calculated using Cox PH Model. Our analysis showed that a combination of ANXA2 expression combined with the Gleason score can be used for predicting the OS of prostate cancer patients.
